# Control of endothelial tubulogenesis by Rab and Ral GTPases, and apical targeting of caveolin-1-labeled vacuoles

**DOI:** 10.1371/journal.pone.0235116

**Published:** 2020-06-22

**Authors:** Pieter R. Norden, Zheying Sun, George E. Davis

**Affiliations:** Department of Molecular Pharmacology and Physiology, Morsani College of Medicine, University of South Florida, Tampa, FL, United States of America; University of Illinois at Chicago, UNITED STATES

## Abstract

Here, we examine known GTPase regulators of vesicle trafficking events to assess whether they affect endothelial cell (EC) lumen and tube formation. We identify novel roles for the small GTPases Rab3A, Rab3B, Rab8A, Rab11A, Rab27A, RalA, RalB and caveolin-1 in co-regulating membrane trafficking events that control EC lumen and tube formation. siRNA suppression of individual GTPases such as Rab3A, Rab8A, and RalB markedly inhibit tubulogenesis, while greater blockade is observed with combinations of siRNAs such as Rab3A and Rab3B, Rab8A and Rab11A, and RalA and RalB. These combinations of siRNAs also disrupt very early events in lumen formation including the formation of intracellular vacuoles. In contrast, knockdown of the endocytosis regulator, Rab5A, fails to inhibit EC tube formation. Confocal microscopy and real-time videos reveal that caveolin-1 strongly labels intracellular vacuoles and localizes to the EC apical surface as they fuse to form the luminal membrane. In contrast, Cdc42 and Rab11A localize to a perinuclear, subapical region where intracellular vacuoles accumulate and fuse during lumen formation. Our new data demonstrates that EC tubulogenesis is coordinated by a series of small GTPases to control polarized membrane trafficking events to generate, deliver, and fuse caveolin-1-labeled vacuoles to create the apical membrane surface.

## Introduction

Critical steps during early vasculogenesis and endothelial cell (EC) lumen formation include both the establishment of asymmetric cytoskeletal polarization (i.e. modified tubulins subapically and F-actin basally) and the trafficking of pinocytic intracellular vacuoles or other intracellular vesicles to create an apical membrane surface[1–16]. Importantly, the asymmetric distribution of cytoskeletal components serves a critical function for organization of membrane trafficking events within ECs and other tubulogenic cells[11–14, 17–19]. In addition, recent work from our laboratory has demonstrated a necessary role for the subapical distribution of modified tubulins (i.e. acetylated tubulin and detyrosinated tubulin) to direct intracellular vacuoles toward a polarized, perinuclear location where they coalesce together to form the luminal membrane[11–14]. These processes are controlled by the activity of small GTPases (including Cdc42, Rac2, k-Ras, and Rap1b), their downstream effectors, and Src family kinases[3, 7, 8, 13, 14, 20]. A central question is how intracellular vacuolization, directed trafficking of vacuoles and vesicles along an asymmetrically polarized cytoskeleton, and coalescence of vacuoles/vesicles to form the lumen are spatially and temporally controlled by molecular regulators of membrane trafficking events, including Rab, Ral, and Rho GTPases.

ECs and other cell types are known to transport membrane compartments through endocytic mechanisms including clathrin-dependent, clathrin-independent, caveolae/caveolin-1 dependent, as well as macropinocytic and phagocytic mechanisms[21–26]. Membrane fusion events are key to deliver cargo to different intracellular compartments or the extracellular space through exocytic mechanisms[23, 27, 28]. The Rab family of GTPases comprise a large family of small GTPases that are major regulators of membrane trafficking and fusion events in eukaryotic cells[28–30]. Recent work using epithelial models of lumen morphogenesis have identified a critical role for Rab11A-Rab8A activity in mediating trafficking of vesicles to apical membrane initiation sites and Rab27/Rab3/Rab8 regulate vesicle tethering and fusion during these events[17, 18, 31–34]. In ECs, Rab3B, Rab3D, Rab27A and the Ras family GTPase RalA have been found to associate with exocytic secretory granules known as Weibel-Palade bodies that are released from ECs to regulate thrombosis and inflammation[35–37]. Interestingly, it was shown that immunostaining of von Willebrand Factor, a component of Weibel-Palade bodies, labeled intracellular vacuole compartments during EC lumen formation[1], implicating a role for the above exocytic GTPase regulators during EC lumen morphogenesis. Caveolin-1 is also highly expressed in ECs and has been shown to regulate capillary morphogenesis *in vitro* and *in vivo*[21, 26, 38, 39]. Additionally, caveolae in ECs have been identified to associate with key regulators of EC morphogenesis such as Src, Yes, Raf, Mek, Ras and Rap1[21]. Together, these results implicate a potential role for Rab and Ral GTPases and Caveolin-1 in regulating membrane trafficking events during EC lumen formation and tubulogenesis although the details concerning their specific roles remain unclear.

In this study, we present novel information characterizing the role of regulators of membrane trafficking events during EC lumen formation and tubulogenesis. Our work has identified new roles for the GTPases Rab3A, Rab3B, Rab8A, Rab11A, Rab27A, RalA and RalB as well as Caveolin-1 during this process and siRNA suppression of individual and combinations of these GTPases markedly impairs the ability of ECs to undergo both vacuolation and tubulogenesis. Furthermore, we show that intracellular vacuoles are highly enriched in Caveolin-1, Src family kinases and RalA which then proceed to target to the apical membrane surface while the small GTPases Cdc42, Rab11A and Rab27A are localized in a polarized, subapical region. Thus, EC lumen and tube morphogenesis are controlled by sequential events regulating intracellular vacuole generation, polarized vesicle trafficking and fusion that occurs in coordination with key small GTPases, signaling pathways and cytoskeletal polarization to control how ECs form cell-lined tube networks in 3D extracellular matrix environments.

## Materials and methods

### Reagents

Stem cell factor (SCF), stromal cell-derived factor 1 alpha (SDF-1α) and interleukin-3 (IL-3) were obtained from R&D systems (Minneapolis, MN). Chemical inhibitors PP3, EHT 1864, and Nexinhib20 were obtained from Tocris (Minneapolis, MN). Ascorbic acid and 12-*O*-tetradecanoyl-phorbol-13-acetate (TPA) were obtained from Sigma-Aldrich (St. Louis, MO). MBQ-167 was obtained from Selleckchem (Houston, TX). Recombinant fibroblast growth factor 2 (FGF-2), antibody against β-Actin and the chemical inhibitors PP2 and GM6001 were obtained from EMD Millipore (Billerica, MA). Antibodies against Rab3B, Rab3D, Rab11A and Rab27A were obtained from Abcam (Cambridge, MA). Antibodies against Caveolin-1, Rab3A, Rab5 and Rab8 were obtained from Cell Signaling Technology (Danvers, MA). An antibody against RalA was obtained from BD Biosciences (San Jose, CA). GFP-RalA and S-Ch-Cdc42 adenoviruses were generated and utilized as described previously[13, 40].

### Vasculogenic tube assembly assays

Human umbilical vein endothelial cells were obtained from Lonza (Walkersville, MD) and were cultured (passage 3–6) as described previously[40].

For use in siRNA-mediated knockdown assays, siRNA transfected ECs were harvested and then suspended at 2 x 10^6^ cells/mL in 2.5 mg/mL collagen type I matrices and assays were performed as described previously[40, 41]. In brief, SCF, IL-3, SDF-1α and FGF-2 were added at 200 ng/mL directly into collagen type I gels[41]. Cultures were then fed with media containing reduced serum supplement II (RSII), ascorbic acid and FGF-2 at 40 ng/mL. Cultures were allowed to assemble into capillary networks for 72 hours, and then cultures were fixed in 3% glutaraldehyde in PBS or collected for additional processing. Following fixation, cultures were then stained in 0.1% toluidine blue in 30% methanol for use in imaging and statistical analysis.

For use in confocal microscopy imaging analysis, ECs were first infected with recombinant adenoviruses as described previously[3]. Infected ECs were then harvested and suspended at 2 x 10^6^ cells/mL in 3.75 mg/mL collagen type I matrices and assays were performed as described previously[42]. In brief, cultures were fed with media containing RSII, ascorbic acid, FGF-2 at 40 ng/mL and TPA at 50 ng/mL[40]. ECs assembled into tube networks over a period of 0–48 hours when cultures were fixed in 3% paraformaldehyde in PBS at the indicated time points. Fixed cultures were then stained before use in confocal fluorescent microscopy imaging.

### EC siRNA suppression

siRNA protocols using the siRNA list below were performed as described previously[40]. ECs were allowed to recover for 48 hrs and the transfection was repeated. ECs were then allowed to recover overnight before being harvested for use in 3D assays or collected for protein expression analysis using standard western blotting techniques.

siRNAs from Ambion are as follows:

Control (AM4637) Silencer Select Negative Control #2

Rab3A (s11666) 5’-CCAUCACCACCGCAUACUA-3’

Rab3B (s11669) 5’-GCUUCAUUCUGAUGUAUGA-3’

Rab3D (s18326) 5’-AGGAGAACAUCAAUGUGAA-3’

Rab5A (s11678) 5’-GGAAGAGGAGUAGACCUUA-3’

Rab8A (s8679) 5’-GCAAGAGAAUUAAACUGCA-3’

Rab11A (s16703) 5’-GAGAUUUACCGCAUUGUUU-3’

Rab27A (s11693) 5’-GCCUCUACGGAUCAGUUAA-3’

RalA (s11758) 5’-GGACUACGCUGCAAUUAGA-3’

RalB (s11762) 5’-CAUGAAUCCUUUACAGCAA-3’

Caveolin-1 (s2446) 5’-GCUUCCUGAUUGAGAUUCA-3’

### Generation of recombinant adenoviruses

Rab11A and Rab27A were amplified from cDNA obtained from Missouri S&T cDNA Resource center (Rolla, MO) and mCherry-Rab5A and sfGFP-Caveolin1 fusion constructs were amplified from cDNA obtained from Addgene (Cambridge, MA) and Src was amplified from cDNA obtained from Open Biosystems (Open Biosystems, GE Dharmacon, Lafayette, CO). Standard restriction digest cloning protocol was used to subclone amplified Rab11A and Rab27A into pmCherry-C1 plasmid and Src into pAcGFP-N1 plasmid (Clontech, Mountain View, CA) using *EcoRI-HF* and *BamHI-HF* and *XhoI* and *AgeI-HF* restriction enzymes respectively (New England Biolabs, Ipswich, MA). Amplified S-epitope (S) tagged S-Ch-Rab5A, S-Ch-Rab11A, S-Ch-Rab27A, Src-GFP-S and sfGFP-Caveolin1 constructs were subcloned into pShuttle-CMV expression plasmid using *NotI-HF* and *XbaI*, *SalI-HF* and *NotI-HF* and *KpnI-HF* and *NotI-HF* restriction enzymes respectively (New England Biolabs). Recombinant adenoviral vectors were then generated and propagated as previously described[3]. The PCR primers used are listed below with the upstream primer first followed by the downstream primer.

Rab11A 5’-AGGAATTCTATGGGCACCCGCGACGACGAGTAC-3’

5’-AGGGATCCTTAGATGTTCTGACAGCACTGCAC-3’

Rab27A 5’-AGGAATTCTATGTCTGATGGAGATTATGATTACC-3’

5’-AGGGATCCTCAACAGCCACATGCCCCTTTCTCC-3’

Src 5’-AGCTCGAGATGGGTAGCAACAAG-3’

5’-AGACCGGTATGAGGTTCTCCCCG-3’

Primers used for subcloning into the pShuttle-CMV plasmid are as follows. The first primer (S-Cherry) is an upstream primer followed by downstream primers for S-Ch-Rab5A, S-Ch-Rab11A and S-Ch-Rab27A. The following sets are pairs of upstream and downstream primers for Src-GFP-S and sfGFP-Caveolin1 respectively.

S-Cherry 5’-AGGCGGCCGCACCATGGCAAAAGAAACCGCTGCTGCGAAATTTG

AACGCCAGCACATGGACTCGATGGTGAGCAAGGGCGAGGAG-3’

Rab5A 5’-AGTCTAGATTAGTTACTACAACACTGATTCCTGGTTGGC-3’

Rab11A 5’-AGTCTAGATTAGATGTTCTGACAGCACTGCAC-3’

Rab27A 5’-AGTCTAGATCAACAGCCACATGCCCCTTTCTCC-3’

Src-GFP-S 5’-AGGTCGACATGGGTAGCAACAAGAG-3’

5’- AGGCGGCCGCTTACGAGTCCATGTGCTGGCGTTCAAATTTCGCAGCAG

CGGTTTCTTTACCAGACTTGTACAGCTCATCCATGCCGTG-3’

sfGFP-Caveolin1 5’-AGGGTACCACCATGGTGAGCAAGGGCGAGGAGCTGTTC-3’

5’-AGGCGGCCGCTTATATTTCTTTCTGCAAGTTGATGCGG-3’

### Microscopic imaging and analysis

Fluorescent imaging of 3D EC cultures undergoing tube formation was performed using a confocal microscope (Leica TCS SPE, Leica, Buffalo Grove, IL) with excitation wavelengths of 405 and 488 or 405, 488 and 561 nm sequentially. We used a 63X water immersion objective (NA 1.2) to capture high-resolution images and in addition, Leica application suite advanced fluorescence (LAS-AF) software was utilized to obtain and process images. 3D endothelial cultures were stained with toluidine blue and imaged using light microscopy and inverted microscopes (Eclipse TE2000-E; Nikon, Melville, NY with Photometrics CoolSNAPHQ2 camera, Tucson, AZ, and Olympus CKX41 with Olympus DP70 camera, Center Valley, PA). EC culture photographs were analyzed using Metamorph software (Molecular Devices, Sunnyvale, CA) that allowed for the quantification of vessel and lumen areas.

### Statistical analysis

Statistical analysis of selected EC vasculogenic and lumen formation data was performed using Microsoft Excel (Microsoft) or Prism 8 (GraphPad). Statistical significance was set at p< 0.05. Student’s t-tests were used when analyzing two groups within a single experiment (with a minimum n = 10 from representative experiments or consolidated experiments and a range from n = 10 to n = 36). In all cases, each experimental condition was performed in triplicate and data points were derived from representative images from each of these wells. ANOVA with post-hoc Tukey analysis was performed when multiple comparisons between samples were evaluated.

## Results

### Regulators of vesicle trafficking control EC tubulogenesis in 3D collagen matrices

**Fig 1:**
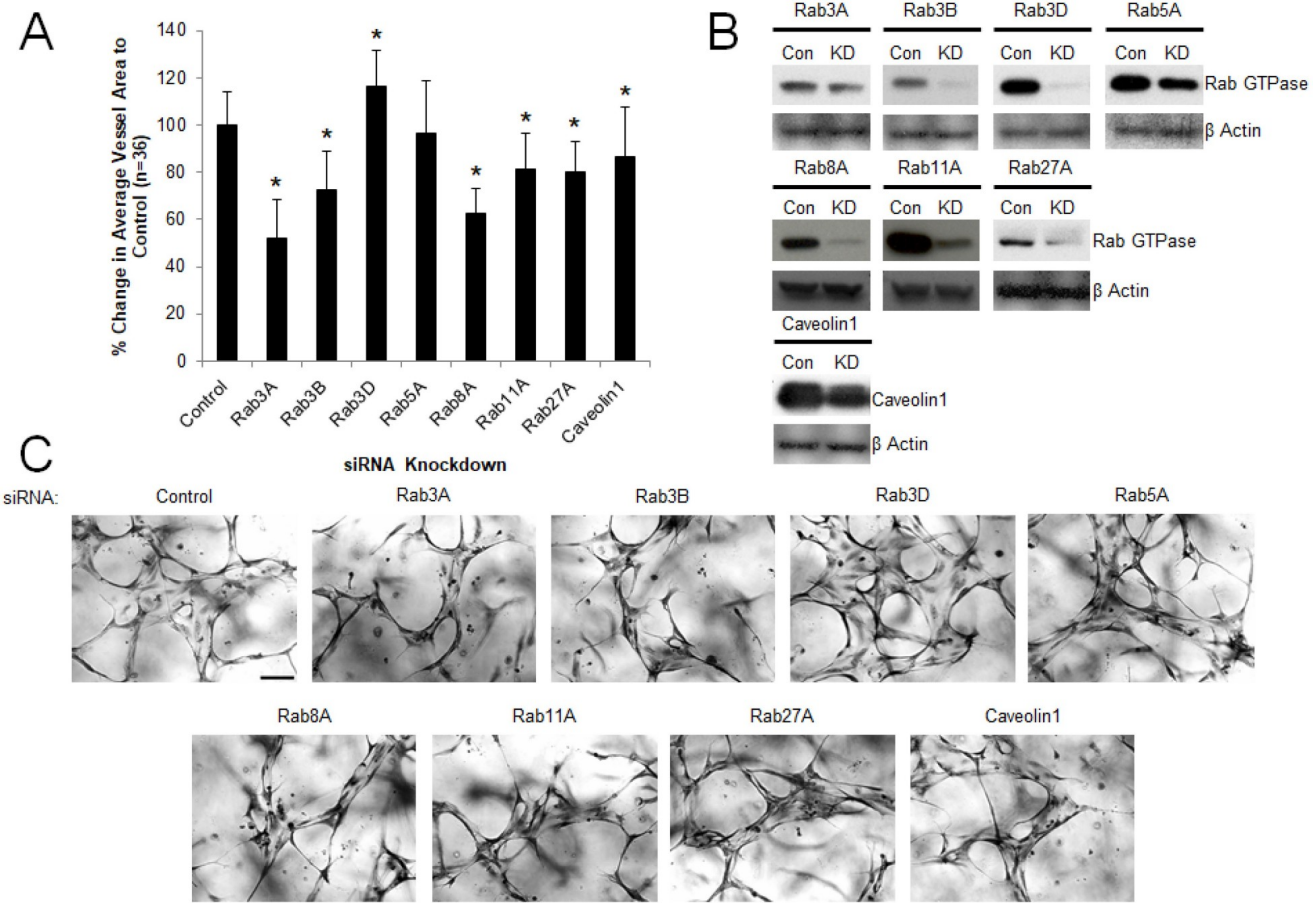
Identification of key membrane trafficking regulators controlling EC tubulogenesis in 3D matrices. (A) EC cultures were transfected with control siRNA or siRNA directed to Rab3A, Rab3B, Rab3D, Rab5A, Rab8A, Rab11A, Rab27A and Caveolin-1, and then were suspended in 3D collagen matrices for 72 hr. before fixation, staining, and photography. Data are normalized to control samples and are reported as average vessel area per high-powered field (HPF) ± standard deviation (SD) (n = 36, p < .05). Asterisk indicates significance compared to control cultures. (B) Lysates generated from transfected cultures in (A) were used in Western blots to assess specific protein knockdown versus control. (C) Representative images from transfected cultures in (A) are shown. Bar equals 100 μm.

**Fig 2:**
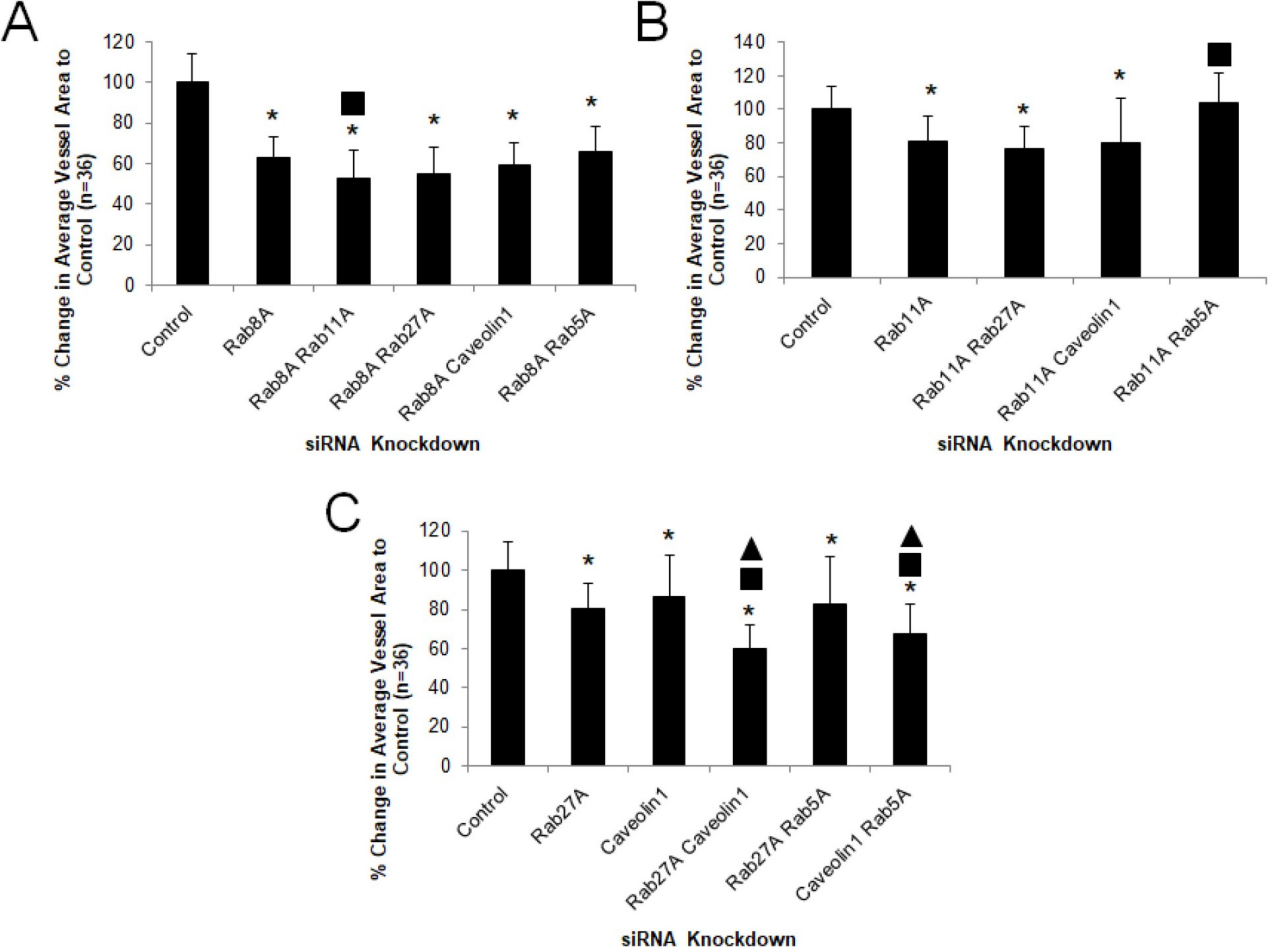
Combined siRNA suppression of Rab GTPases and Caveolin-1 reveal key functional roles during EC tubulogenesis. Individual EC cultures were transfected with the indicated siRNAs singly or in combination and suspended in 3D collagen matrices for 72 hr before being fixed, stained, photographed and quantified (A,B,C). Data are normalized to control samples and are presented as average vessel area per HPF ± SD (n = 36). Asterisks indicate significance at p < .05 to control whereas squares indicate significance at p < .05 to Rab8A (A), Rab11A or caveolin-1 (B), and triangles indicate significance at p < .05 to Caveolin-1 (C).

**Fig 3:**
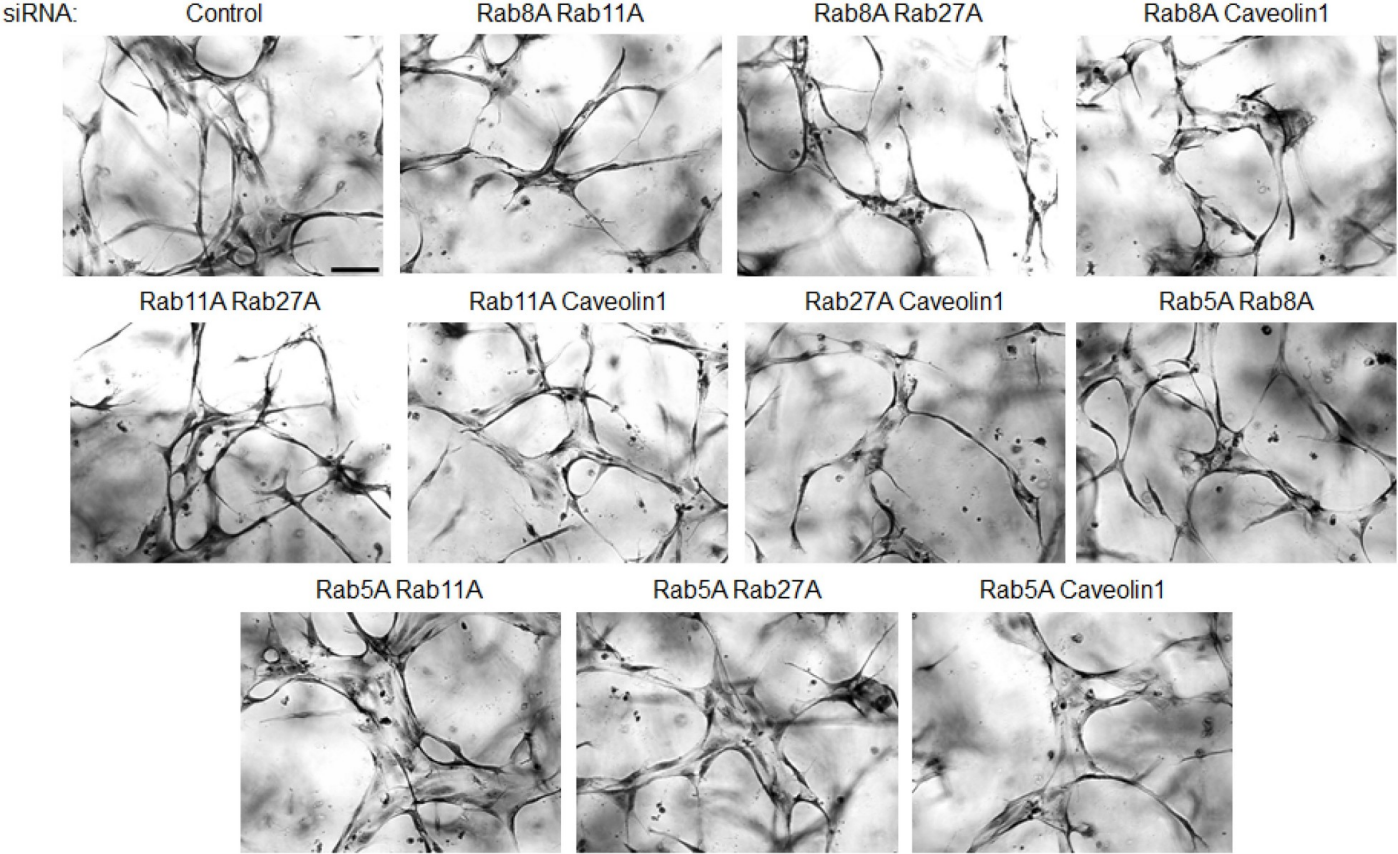
EC tubulogenesis requires membrane trafficking events regulated by Rab8A, Rab11A, Rab27A and Caveolin-1. ECs were transfected with control siRNA or the indicated combinations of siRNAs and suspended in 3D collagen matrices for 72 hr. before being fixed, stained and photographed. Representative images of cultures are shown. Bar equals 100 μm.

Our previous work suggests a key role for small GTPases such as Cdc42, Rac1, Rac2, k-Ras, and Rap1b, and their downstream effectors such as Pak2, Pak4 and Rasip-1, in controlling EC lumen formation[3, 7, 8, 13, 16, 43]. To address a potential role of known key regulators of vesicle trafficking in ECs and other cell types, we performed a screen of Rab GTPases and Caveolin-1 during EC tubulogenesis by siRNA suppression and identified several new regulators of these events (**Fig 1**). Our data implicates a key role for Rab3A, Rab3B, Rab8A, Rab11A, Rab27A and Caveolin-1 during this process where Rab3A and Rab8A knockdown have the most marked effects. In contrast, Rab5A knockdown showed no effects whereas Rab3D may be inhibitory as knockdown stimulated EC tubulogenesis (**Fig 1**). Using combinations of siRNAs, we demonstrated that combined knockdown of Rab8A with Rab11A or Rab27A had a greater blocking effect compared to knockdown of Rab8A alone and that combined knockdown of Rab27A and Caveolin-1 had a greater blocking influence than knockdown of Rab27A or Caveolin-1 alone. Furthermore, these combined knockdowns had the greatest blocking effect compared to other siRNA combinations tested (**Figs 2 and 3**). This data suggests that vesicle trafficking events controlled by Rab3A, Rab3B, Rab8A, Rab11A, Rab27A and Caveolin-1 appears to be necessary for ECs to form lumens and assemble into capillary networks in 3D matrix environments whereas, the endosomal trafficking regulator, Rab5A, does not appear to be involved.

### The exocytosis regulators RalA and RalB control EC tubulogenesis

**Fig 4:**
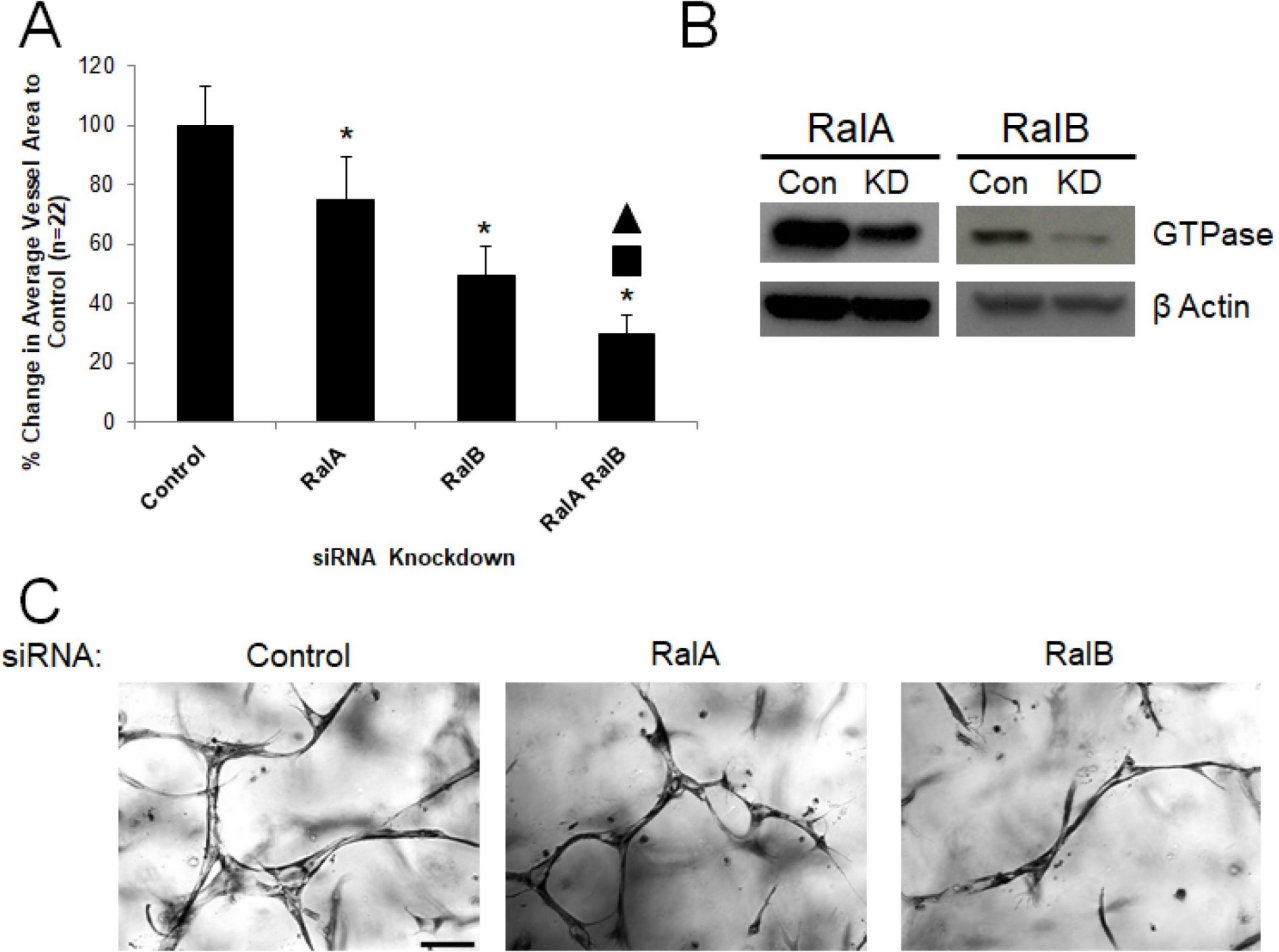
RalA and RalB are required for EC tubulogenesis in 3D matrices. (A) EC cultures were transfected with a control siRNA or siRNA directed to RalA or RalB and cultures were suspended in 3D collagen matrices for 72 hr. before being fixed, stained, photographed and quantified. Data are normalized to control samples and are reported as average vessel area per HPF ± SD (n = 22, p < .05). Asterisks indicate significance to control samples, while the square and triangle indicate significance to RalA and RalB, respectively. (B) Lysates generated from transfected cultures in (A) were used in Western blots to assess specific protein knockdown versus control. (C) Representative images from transfected cultures in (A) are shown. Bar equals 100 μm.

**Fig 5:**
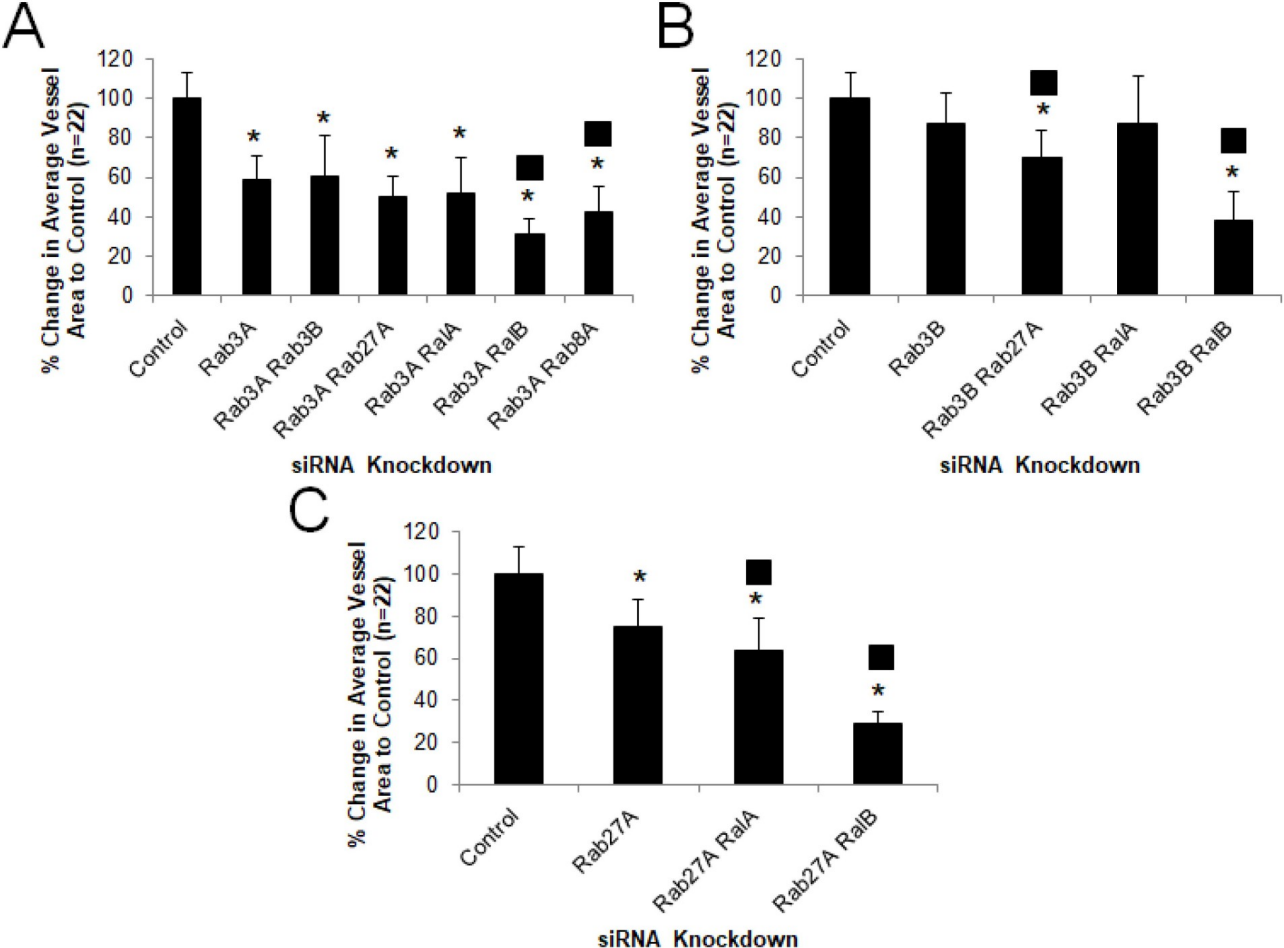
Combinatorial control of EC tubulogenesis by Rab and Ral GTPases in 3D matrices. Individual EC cultures were transfected with the indicated siRNAs singly or in combination and suspended in 3D collagen matrices for 72 hr before being fixed, stained, photographed and quantified (A,B,C). Data are normalized to control samples and are presented as average vessel area per HPF ± SD (n = 22). Asterisks indicate significance at p < .05 to control whereas squares indicate significance at p < .05 to Rab3A (A), Rab3B (B) or Rab27A (C).

**Fig 6:**
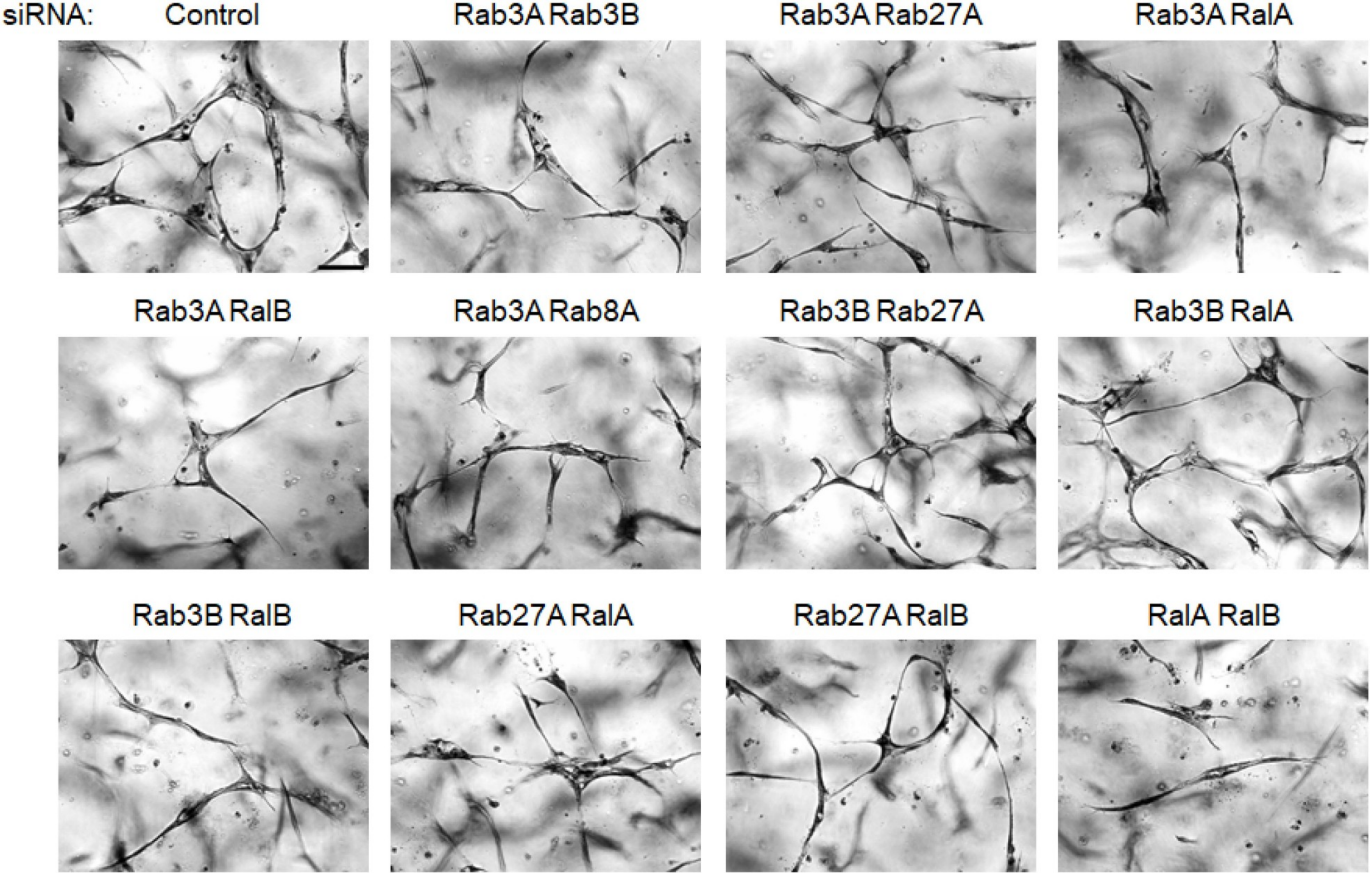
Critical roles for Rab3A, Rab3B, Rab27A, RalA, and RalB during EC tubulogenesis. ECs were transfected with control siRNA or the indicated combinations of siRNAs and suspended in 3D collagen matrices for 72 hr before being fixed, stained and photographed. Representative images of cultures are shown. Bar equals 100 μm.

Because we observed strong blocking effects on tubulogenesis with siRNA suppression of Rab3A, Rab3B and Rab27A (which all play a role in exocytosis) (**Fig 1**), we addressed the role of both isoforms of the Ral GTPases, which are known to regulate exocytosis through their association with the exocyst complex[44], and with Weibel-Palade bodies in ECs[45]. siRNA suppression of either RalA or RalB blocked EC tubulogenesis with a more prominent effect observed with knockdown of RalB (**Fig 4**). Using additional combinations of siRNAs, we show that combined knockdown of RalB with Rab3A, Rab3B, Rab27A, and RalA had the greatest blocking effects compared to any of the blocking effects observed with individual siRNAs (**Figs 5 and 6**). We also show that combined siRNA suppression of Rab3A and Rab8A has a greater blocking effect than suppression of Rab3A alone (**Figs 5 and 6**). Together, this data suggests that RalA and RalB are critical regulators of EC lumen morphogenesis. The combined influence of RalB siRNA knockdown with other GTPases associated with the regulation of exocytosis also supports the idea that these molecules play key functional roles during lumen formation. Furthermore, our results from combined siRNA suppression of Rab3A and Rab8A demonstrates a possible role for regulation of differing endocytic and exocytic membrane vesicle trafficking steps during EC tubulogenesis.

### Vesicle trafficking regulators play an early role in the creation of EC lumens by affecting intracellular vacuole formation

**Fig 7:**
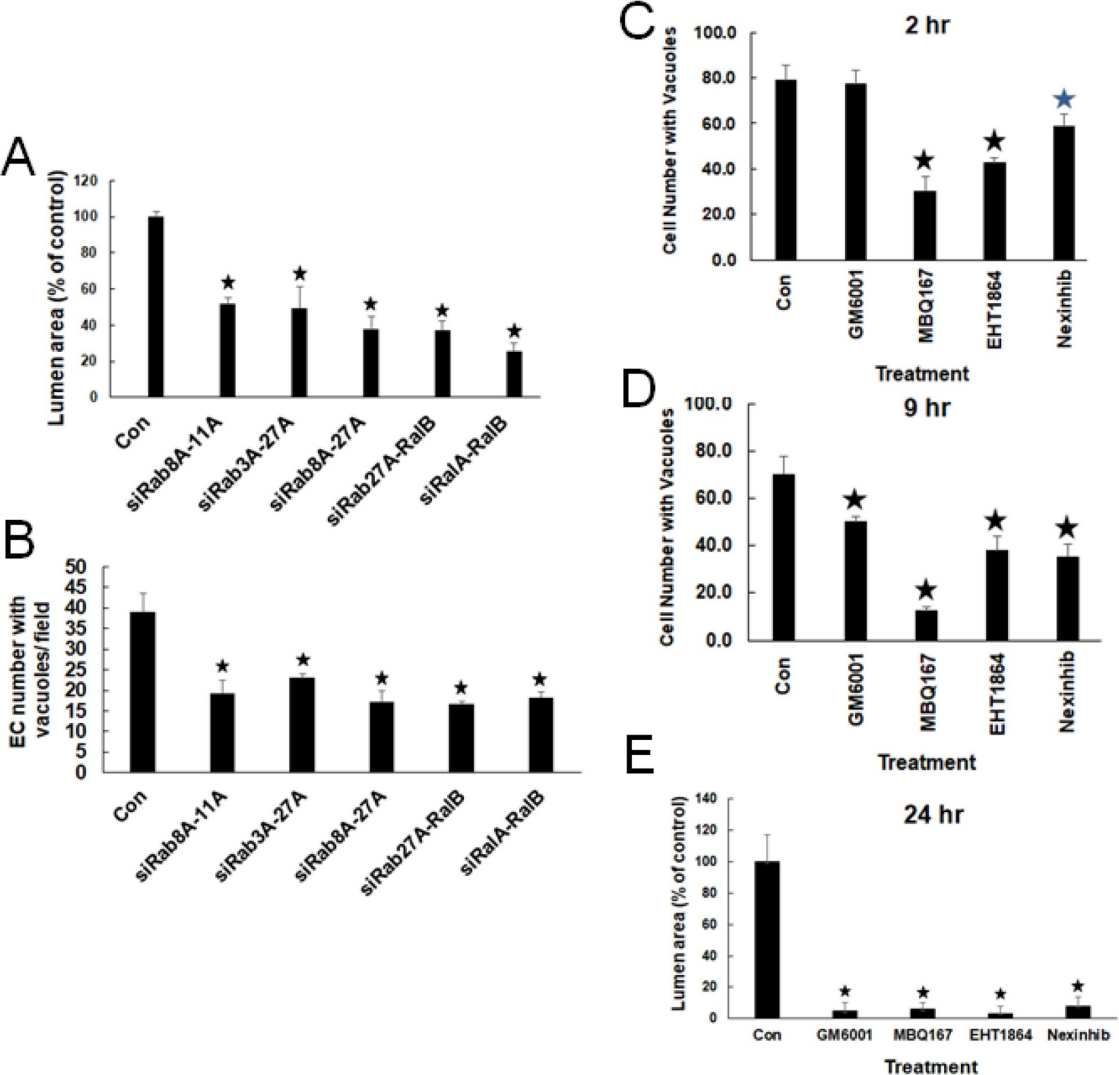
Key vesicular trafficking regulators control lumen formation in part by affecting the generation of intracellular vacuoles. (A,B) ECs were treated with the indicated combinations of siRNAs and were evaluated at either 24 hr (A) for lumen area or 6 hr of culture for ECs with multiple intracellular vacuoles per field (B). Asterisks indicate significance at p< .05. (C,D) ECs were treated with the indicated pharmacologic inhibitors vs. control and cultures were fixed and quantitated for vacuole formation at either 2 or 9 hr. The inhibitors were added at the following doses: GM6001 (10 μM), MBQ167 (2.5 μM), EHT1864 (25 μM), and Nexinhib (6.25 μM). (E) The same inhibitors and doses were added to EC cultures to assess their influence on lumen formation which was quantitated after 24 hr. Asterisks indicate significance at p < .05. Each of the data points is derived from triplicate wells (n = 12) from a representative experiment.

Our previous work demonstrates a major role for intracellular vacuole formation as a key initiation step in stimulating the EC lumen formation process. To address whether we can detect differences in the temporal role of vesicle trafficking regulators vs. previously identified regulators of EC intracellular vacuole and lumen formation, we performed siRNA suppression and pharmacologic inhibition experiments. We focused on combinations of siRNAs that had strong inhibiting effects on lumen formation (**Fig 7A**), and these siRNAs also similarly blocked intracellular vacuole formation (**Fig 7B**). We anticipated that there would be distinctions between them, but there was not, demonstrating that these different classes of vesicle trafficking regulators, have overlapping functions in regulating both early and later steps in the lumen formation process.

**Fig 8:**
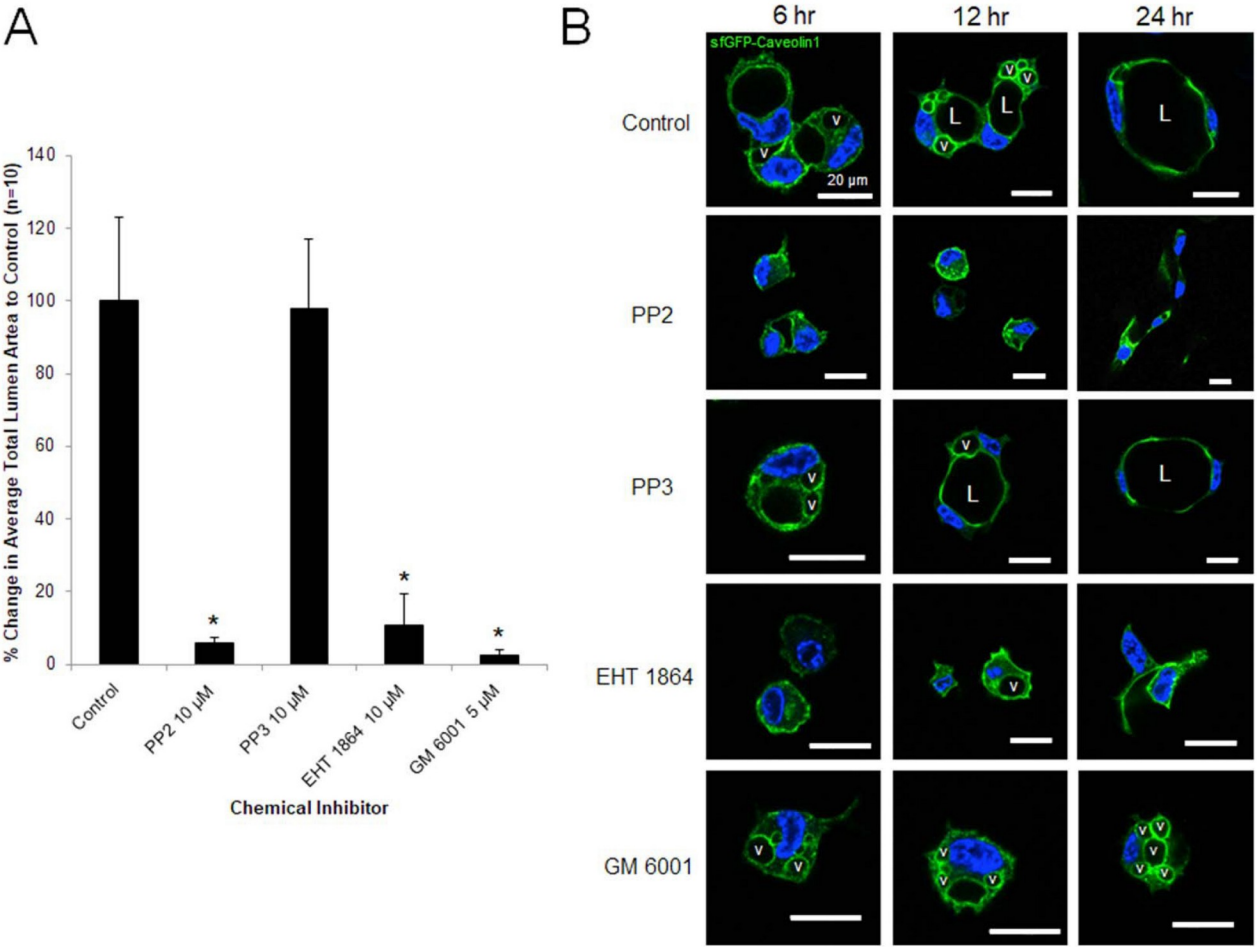
Src and Rac activities generate GFP-caveolin-1 labeled intracellular vacuoles that are necessary for EC tubulogenesis while MT1-MMP activity regulates downstream fusion of intracellular vacuoles to control EC lumen formation. (A) Cultured ECs were harvested, suspended in 3D collagen matrices and fed with control culture media or media containing either PP2 10 μM, PP3 10 μM, EHT 1864 10 μM or GM6001 5 μM and allowed to form vacuoles and lumens for 24 hr before being fixed, stained, photographed and quantified for lumen area. Data are normalized to control samples and are reported as average lumen area per HPF ± SD (n = 10, p < .05). Asterisk indicates significance to control samples. (B) ECs cultures were infected with recombinant adenovirus carrying sfGFP-Caveolin1 and treated with control culture media or culture media containing chemical inhibitors as in (A) and, were allowed to form vacuoles and lumens over a period of 6–24 hr. Fixed cultures were then analyzed by confocal microscopy and representative images are shown. L indicates the EC lumen space and v indicates vacuoles. Bar equals 20 μm.

We also tested pharmacologic inhibitors for a comparison, and interestingly, the Rab27A inhibitor, Nexinhib20, blocked lumen formation, but also intracellular vacuole formation at different early time points during EC lumen formation (**Fig 7C–7E**). These results are similar to that observed with siRNA combinations including Rab27A siRNA (**Fig 7A and 7B**). In addition, we compared these results to addition of Cdc42 and Rac inhibitors such as MBQ-167 (Cdc42 and Rac inhibitor), and EHT1864 (Rac inhibitor) which interfere with both EC intracellular vacuole and lumen formation (**Fig 7C–7E**). Interestingly, an inhibitor of MT1-MMP, GM6001, strongly blocks lumen formation, but has less influence on intracellular vacuole formation (**Figs 7 and 8**). Our data does suggest that lumen expansion requires MT1-MMP and this appears to require intracellular vacuole-vacuole fusion events. We observe retention of EC intracellular vacuoles (labeled with GFP-caveolin-1) during lumen formation in the presence of GM6001, particularly observed later in the process (**Fig 8**). Initial vacuole formation is not inhibited by GM6001, but further lumen formation is markedly inhibited, although vacuoles are retained over time in the GM6001-treated ECs, but not in control ECs. In contrast, addition of the lumen formation inhibitors, PP2 (Src inhibitor) and EHT1864, markedly block both vacuole and lumen formation, while PP3, an inactive control for PP2, has no effect (**Fig 8**). Overall, these data indicate a complex and overlapping role for Rab and Ral GTPases in controlling both early (through intracellular vacuole formation) and later phases of lumen formation (vacuole-vacuole fusion and lumen expansion events).

### Caveolin-1-, RalA-, and Src-enriched intracellular vacuoles are trafficked from the basal membrane surface to form the apical luminal membrane during EC tubulogenesis

**Fig 9:**
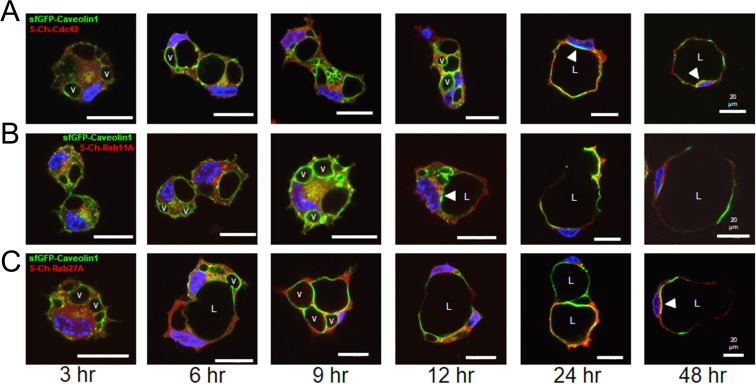
GFP-Caveolin-1 targets to intracellular vacuoles and the apical membrane surface during EC tubulogenesis in 3D matrices, while mCherry- labeled Cdc42, Rab11A, and Rab27A target to a subapical region during EC lumen formation. ECs were infected with recombinant adenovirus carrying sfGFP-Caveolin-1 in combination with recombinant adenoviruses expressing S-Ch-Cdc42 (top row), S-Ch-Rab11A (middle row), or S-Ch-Rab27A (bottom row) and were allowed to form vacuoles and lumens in 3D collagen matrices for 3–48 hr. to visualize this process. Fixed cultures were then analyzed by confocal microscopy and representative images are shown. White arrowheads indicate targeting of Caveolin-1 to the apical membrane surface and subapical distribution of Cdc42, Rab11A or Rab27A. v indicates examples of vacuoles containing Caveolin-1. L indicates the EC lumen space. Bar equals 20 μm.

**Fig 10:**
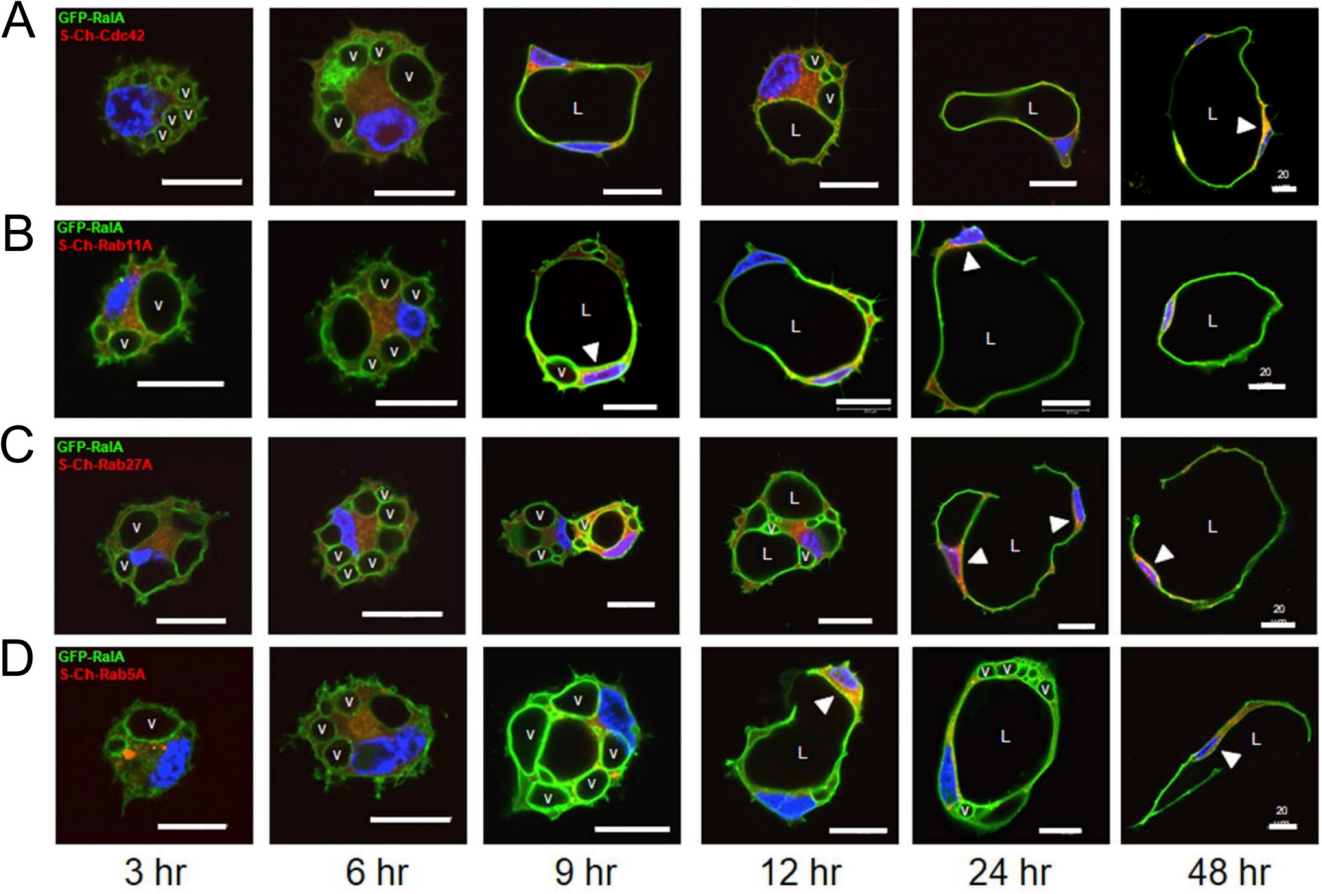
GFP-RalA targets to intracellular vacuoles and the apical membrane surface during EC tubulogenesis in 3D matrices. ECs were infected with recombinant adenovirus carrying GFP-RalA in combination with recombinant adenoviruses expressing S-Ch-Cdc42 (first row), S-Ch-Rab11A (second row), S-Ch-Rab27A (third row) or S-Ch-Rab5A (fourth row) and, were allowed to form vacuoles and lumens in 3D collagen matrices for 3–48 hr. to visualize this process. Fixed cultures were then analyzed by confocal microscopy and representative images are shown. White arrowheads indicate targeting of Ral-A to the apical membrane surface and subapical distribution of Cdc42, Rab11A, Rab27A or Rab5A. v indicates examples of vacuoles containing RalA. L indicates the EC lumen space. Bar equals 20 μm.

**Fig 11:**
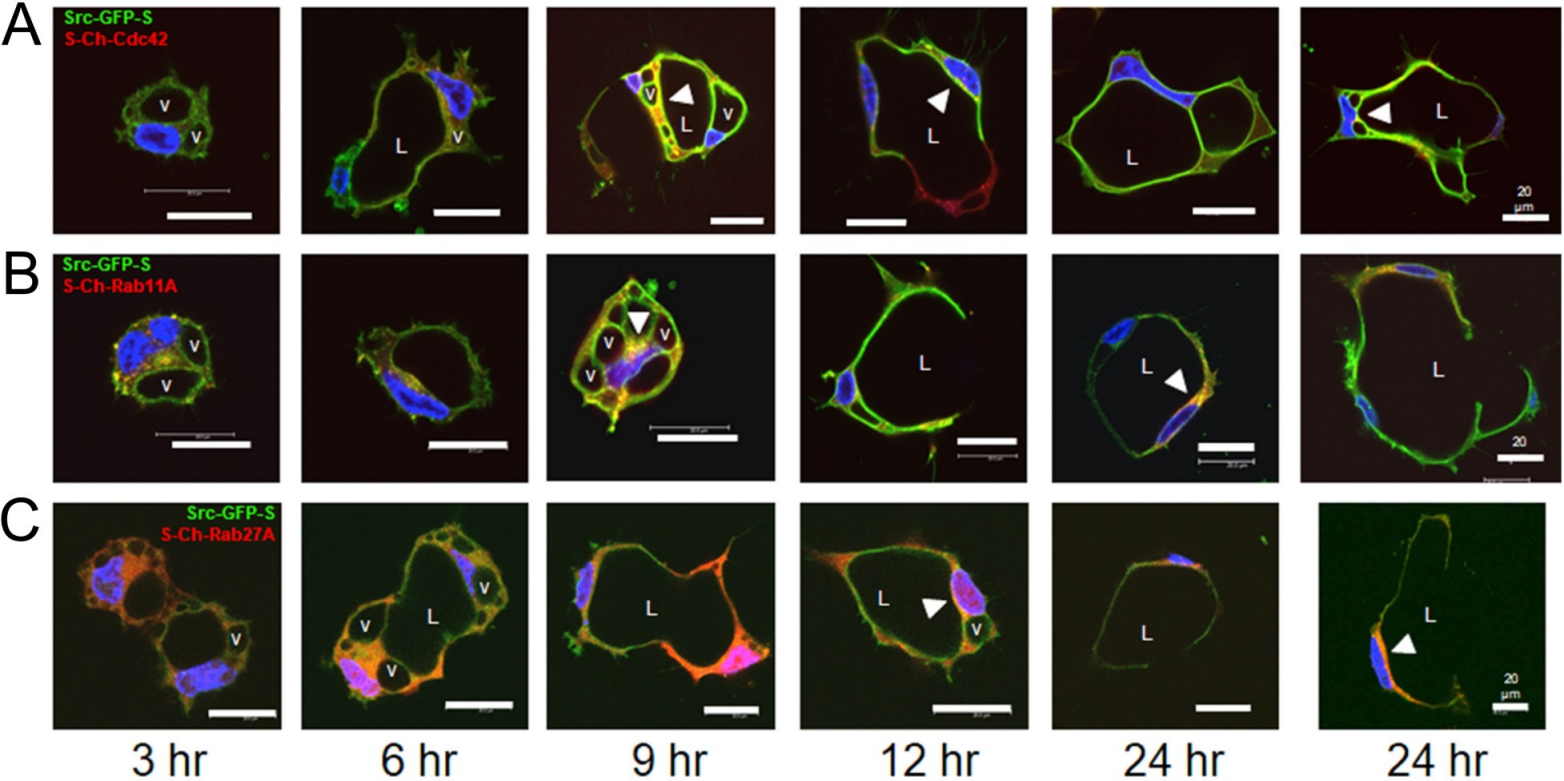
Src-GFP targets to intracellular vacuoles and the developing apical membrane surface during EC tubulogenesis in 3D matrices. ECs were infected with recombinant adenovirus carrying Src-GFP-S in combination with recombinant adenoviruses expressing S-Ch-Cdc42 (top row), S-Ch-Rab11A (middle row), or S-Ch-Rab27A (bottom row) and, were allowed to form vacuoles and lumens in 3D collagen matrices for 3–24 hr. to visualize this process. Fixed cultures were then analyzed by confocal microscopy and representative images are shown. White arrowheads indicate subapical distribution of Src and Cdc42, Rab11A or Rab27A. v indicates examples of vacuoles containing Src. L indicates the EC lumen space. Bar equals 20 μm.

To investigate the subcellular localization of additional key regulators of EC lumen formation and molecules associated with membrane trafficking events, we generated a panel of recombinant adenovirus reagents expressing mCherry-fusion or GFP-fusion proteins. We then used multiple combinations of these reagents to assess whether these molecules co-localize with one another over time during different stages of EC lumen morphogenesis as assessed using real-time movies (**S1–S5 Videos**) and confocal microscopic imaging over time (**Figs 9–11**). Here, by using sfGFP-Caveolin-1 and GFP-RalA labeled ECs, we show that Caveolin-1 (**Fig 9A–9C**) and RalA (**Fig 10A–10D**) strongly localize to intracellular vacuoles generated from the basal membrane surface that are trafficked apically and fuse within a polarized perinuclear region to form the EC apical luminal membrane over time. Real-time videos demonstrate marked targeting of sfGFP-Caveolin-1 and GFP-RalA to intracellular vacuoles and the developing apical surface, while S-mCherry (S-Ch)-Rab11A, S-Ch-Cdc42 and S-Ch-Rab5A did not (**S1–S5 Videos**). Additionally, using a Src-GFP-S construct we show that Src also targets to intracellular vacuoles during this process (**Fig 11A–11C**). As the intracellular vacuoles coalesce to form the lumen during later events, we also show that Caveolin-1, RalA, and Src target to the apical membrane surface. In contrast, use of S-Ch-Cdc42, S-Ch-Rab11A, S-Ch-Rab27A and S-Ch-Rab5A constructs showed that Cdc42, Rab11A, Rab27A and Rab5A all show targeting ability to the subapical domain region (**Figs 9–11**). During early EC lumen formation, Rab11A appears to localize to vesicles in a punctate manner that accumulate in a polarized, perinuclear region (**Fig 9B**) whereas Rab27A is initially diffuse throughout the cytoplasm (**Figs 9C and 10C**) and Rab5A is localized to more diffuse, punctate regions (**Fig 10D**). Furthermore, it appears that caveolin-1-, RalA-, and Src-strongly label intracellular vacuoles which are trafficked apically where they accumulate in these polarized, perinuclear regions, that are enriched in Rab11A and Cdc42.

## Discussion

**Fig 12:**
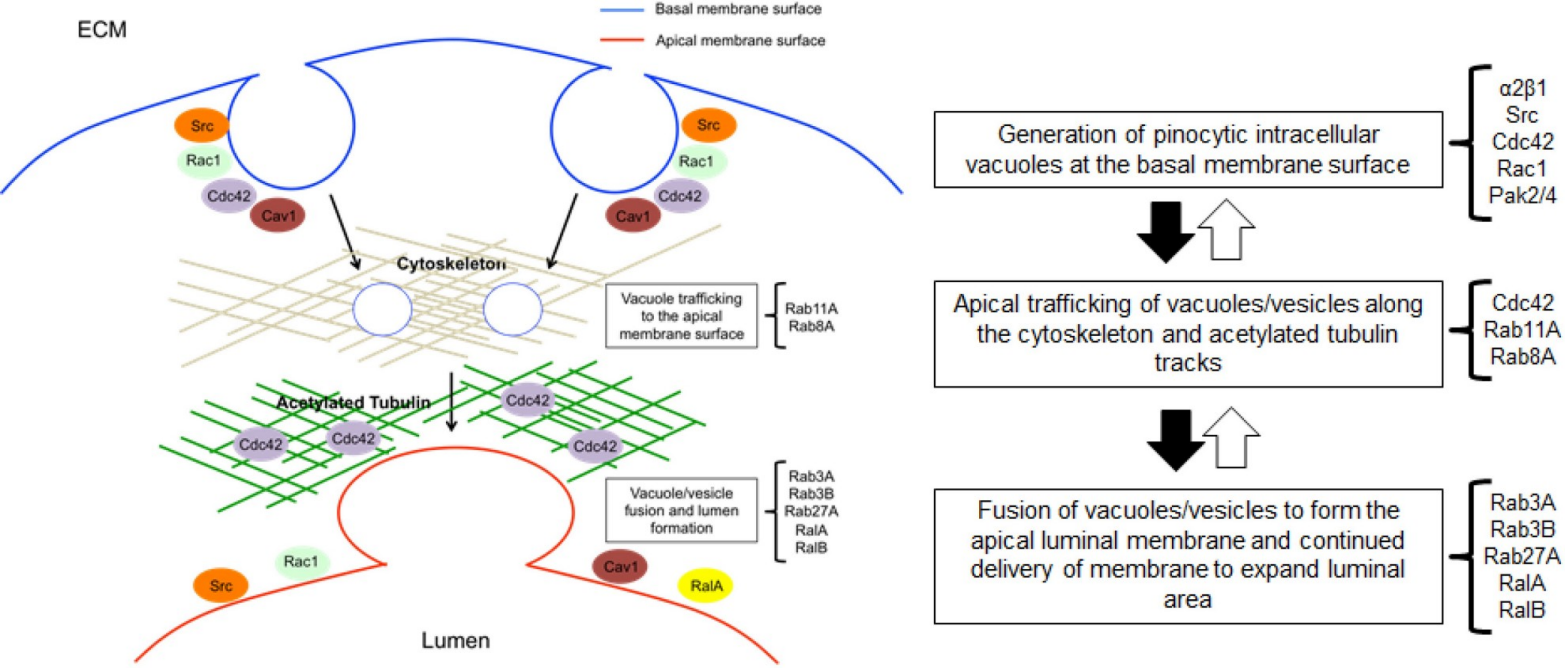
Generation, apical trafficking and fusion of pinocytic intracellular vacuoles along the tubulin cytoskeleton is controlled by Cdc42, Rac1, Rab and Ral GTPases in coordination with Src and Caveolin-1 during EC tubulogenesis. A schematic diagram is shown illustrating major steps in EC lumen formation that are regulated by key small GTPases, Src family kinases and Caveolin-1. Intracellular vacuole membranes containing Src, Rac1, Caveolin-1, and RalA are generated at the basal membrane surface where they are then trafficked apically to a polarized perinuclear location along acetylated tubulin tracks. Vacuole-vacuole fusion occurs to create and expand the luminal space. MT1-MMP plays a key role in EC lumen expansion which facilitates these vacuole fusion events. These vacuole trafficking and fusion events appear to be regulated by the combined action of Rab11A, Rab8A, Rab3A, Rab3B, Rab27A, RalA and RalB.

Intracellular vacuolization is a critical mechanism controlling EC lumen, tube and capillary assembly[1, 3–6, 12–14, 19, 46]. Important to our understanding of this process is to elucidate the molecular mechanisms underlying how ECs generate intracellular vacuoles, traffic them along microtubule cytoskeletal tracks and then for individual vacuoles to fuse together in a polarized perinuclear region to create the apical luminal membrane surface[12–14, 46]. In the work presented here, we identify novel roles for Rab3A, Rab3B, Rab8A, Rab11A, Rab27A, RalA and RalB small GTPases and Caveolin-1 in controlling these EC lumen formation events in coordination with Src, Cdc, Rac1 and MT1-MMP activities (**Fig 12**). We also demonstrate critical roles for key molecular regulators of EC tubulogenesis in controlling apical-basal polarization such as Caveolin1, RalA and Src which markedly target to intracellular vacuoles and the developing apical membrane, while Cdc42, Rab11A and Rab27A localize within a subapical domain. Finally, we demonstrate that generation of intracellular vacuoles is controlled by multiple GTPases including Rab27A, Rab3A, Rab11A, RalA and RalB in addition to Cdc42, Rac, and Src activities which are necessary for EC lumen formation. Interestingly, MT1-MMP, which has much less influence on intracellular vacuole formation appears to have a key downstream function in facilitating vacuole-vacuole fusion events to generate and expand the luminal compartment.

As Rab GTPases are the largest family of small GTPases and regulate a variety of membrane trafficking events[27, 28, 30], they have become an important target of investigation for studying how membrane vesicles are delivered to a nascent apical domain during *de novo* lumen formation. Recent work in Madin-Darby Canine Kidney cyst models of epithelial lumen formation have implicated roles for Rab11A-Rab8A and Rab27/Rab3/Rab8 pathways in controlling *de novo* development of lumen cysts[17, 18, 31, 32]. However, a role for Rab GTPases in regulation of endothelial lumen formation and tubulogenesis has not been previously investigated. Here, we show that siRNA suppression of Rab3A, Rab3B, Rab8A, Rab11A or Rab27A impairs endothelial tubulogenesis in 3D collagen matrices where suppression of Rab3A and Rab8A possess the strongest blocking effects. Additionally, we also show that siRNA suppression of Caveolin-1, RalA and RalB blocks EC tubulogenesis supporting the conclusion that multiple GTPases, as well as Caveolin-1, regulate critical membrane trafficking events necessary for EC tubulogenesis. In contrast, we demonstrate that siRNA suppression of Rab5A had no effect on EC lumen formation, while an siRNA to Rab3D stimulated lumen formation. Our work here does suggest that these GTPases likely work together to control the complex process of EC lumen and tube formation. In support of such a premise, a process such as Weibel-Palade body exocytosis in ECs, involves a series of small GTPase regulators including Rab27A and Rab3A[36, 37, 45, 47]. These conclusions are based on our studies showing that combined siRNA suppression of Rab8A and Rab11A, Rab8A and Rab27A, and Rab27A and Caveolin1 had stronger blocking effects compared to individual suppression of Rab8A, Rab27A, or Caveolin-1. Additionally, we show that combined siRNA suppression of RalB with Rab3A, Rab3B, Rab27A or RalA markedly blocks tubulogenesis, implicating an important role for RalB during this process. Together, our results show similar functional requirements of Rab3A, Rab8A, Rab11A and Rab27A compared to what has been previously reported using MDCK cyst models of epithelial lumen formation. Interestingly, previous studies using this model implicated an ability of Rab11A-Rab8A to act in a signaling pathway that affected the activity of the critical GTPase, Cdc42, to control epithelial lumen formation[18]. Our studies here demonstrating the overlap of key small GTPase control of EC lumen formation involving Cdc42 and Rac isoforms with key Rab and Ral isoforms suggest similarities between EC and epithelial lumen formation mechanisms. Future studies are necessary to elucidate the molecular details for how these key GTPases affect each other to control this EC morphogenic process.

The work presented here identifies new regulators of EC polarity and trafficking of intracellular vacuoles during tubulogenesis. We show that as lumen formation progresses, ECs accumulate intracellular vacuoles generated from the basal membrane surface that are enriched in Caveolin-1, RalA, and Src. Of great interest are previous studies implicating a co-role for these molecules in regulating vesicular trafficking events, inducing membrane curvature in developing vesicles, and activation of caveolae to regulate membrane transport functions in ECs and other cell types[44, 48–50]. Previous work from our laboratory showed that activated Src- and Rac-labeled vacuoles were observed to track along the microtubule cytoskeleton that was strongly labeled with acetylated tubulin[12–14]. The accumulating vacuoles position themselves along these acetylated tubulin tracks in a strongly polarized and perinuclear position[12, 14, 46]. These polarized vacuoles then fuse together to create the apical luminal compartment, which is stabilized by the subapical microtubule cytoskeleton that is modified in part through acetylation and detyrosination[11]. In this manner, EC lumen formation is highly polarized within individual cells. Disassembly of this subapical tubulin cytoskeleton leads to disruption of the apical surface and collapse of EC-lined tube networks[11, 51]. This occurs following addition of microtubule depolymerizing agents or the addition of the pro-regressive cytokines and proinflammatory mediators, IL-1β, TNFα and thrombin[11, 51, 52]. During capillary tube regression in response to these proinflammatory mediators, loss of acetylated tubulin was a key component of a capillary regression signaling signature that was identified[52]. Thus, it appears that the microtubule cytoskeleton is necessary to support the formation of the apical membrane surface, but also to maintain its stability as EC-lined tubes mature. In support of this concept, another recent study found that loss of detyrosinated tubulin in placental microvessels correlated with the development of pre-eclampsia[53], a key disease leading to premature births.

Overall, the work presented here advances our understanding of key regulators of EC tubulogenesis and adds that key vesicle trafficking regulators of the Rab and Ral family coordinate with other key small GTPases including Cdc42, Rac1, Rac2, k-Ras, and Rap1b and their effectors to control this critical and complex process. These new studies further support our longstanding work demonstrating a critical role for intracellular vacuole formation in the EC lumen formation process. In a coordinated manner, these key small GTPases impact the formation of intracellular vacuoles, vacuole-vacuole fusion events, the polarization of these processes within individual ECs via the apical membrane and underlying modified microtubule cytoskeleton, and maturation of the EC apical membrane to create multicellular networks of EC-lined tubes.

## Supporting information

S1 Fig Full length blots for Fig 1B. Control and siRNA knockdown samples for each molecule were loaded side-by-side and in between colored molecular weight markers. Blots were cut along the molecular weight marker lanes and were probed with the indicated antibodies and then secondary antibodies coupled to horseradish peroxidase followed by development with enhanced chemiluminescence reagents. Blots were lined up together and exposed to x-ray film. (A) Two independent knockdown experiments are shown together for each of the indicated molecules. (B) The same blots shown in A were stripped and then probed with anti-actin antibodies.
(TIF)

S2 Fig Full length blots for Fig 1B and Fig 4B. Control and siRNA knockdown samples for each molecule were loaded side-by-side and in between colored molecular weight markers. Blots were cut along the molecular weight marker lanes and were probed with the indicated antibodies and then secondary antibodies coupled to horseradish peroxidase followed by development with enhanced chemiluminescence reagents. Blots were lined up together and exposed to x-ray film. (A) A representative knockdown experiment is shown for each of the indicated molecules. The upper panel and lower panels show different exposures of the same blot. (B) The same blots shown in A were stripped and then probed with anti-actin antibodies. The upper panel and lower panels show different exposures of the same blot.(C) A representative knockdown experiment is shown for RalA. The same blot was stripped and probed with anti-actin antibodies.
(TIF)

S1 Video Intracellular vacuole and apical targeting of GFP-caveolin-1 vs. subapical distribution of mCherry-Rab11A during EC lumen formation. ECs were induced to express sfGFP-caveolin-1 and mCherry-Rab11A and then were seeded in 3D collagen matrices for 24 hr. Large arrows indicate key areas to focus on in the video, while small arrows indicate the developing apical membrane surface. The video is shown at 8 frames/sec.
(MP4)

S2 Video Intracellular vacuole and apical targeting of GFP-RalA vs. subapical distribution of mCherry-Rab11A during EC lumen formation. ECs were induced to express GFP-RalA and mCherry-Rab11A and then were seeded in 3D collagen matrices for 24 hr. Large arrows indicate key areas to focus on in the video, while small arrows indicate the developing apical membrane surface. The video is shown at 8 frames/sec.
(MP4)

S3 Video Intracellular vacuole and apical targeting of GFP-caveolin-1 vs. subapical distribution of mCherry-Cdc42 during EC lumen formation. ECs were induced to express sfGFP-caveolin-1 and mCherry-Rab11A and then were seeded in 3D collagen matrices for 24 hr. Large arrows indicate key areas to focus on in the video, while small arrows indicate the developing apical membrane surface. The video is shown at 8 frames/sec.
(MP4)

S4 Video Intracellular vacuole and apical targeting of GFP-RalA vs. subapical distribution of mCherry-Cdc42 during EC lumen formation. ECs were induced to express GFP-RalA and mCherry-Rab11A and then were seeded in 3D collagen matrices for 24 hr. Large arrows indicate key areas to focus on in the video, while small arrows indicate the developing apical membrane surface. The video is shown at 8 frames/sec.
(MP4)

S5 Video Intracellular vacuole and apical targeting of GFP-caveolin-1 vs. cytoplasmic distribution of mCherry-Rab5A during EC lumen formation. ECs were induced to express sfGFP-caveolin-1 and mCherry-Rab5A and then were seeded in 3D collagen matrices for 24 hr. Large arrows indicate key areas to focus on in the video, while small arrows indicate the developing apical membrane surface. The video is shown at 8 frames/sec.
(MP4)
